# The L.E.A.D. Framework: Using Tools From Evidence-Based Public Health to Address Evidence Needs for Obesity Prevention

**DOI:** 10.5888/pcd9:120157

**Published:** 2012-07-12

**Authors:** Shiriki Kumanyika, Ross C. Brownson, Allen Cheadle

**Affiliations:** Author Affiliations: Ross C. Brownson, Prevention Research Center in St. Louis, Brown School, Washington University in St. Louis, St. Louis, Missouri, and the Division of Public Health Sciences and Siteman Cancer Center, Washington University School of Medicine, St. Louis, Missouri; Allen Cheadle, Center for Community Health and Evaluation, Group Health Research Institute, Seattle, Washington

## Introduction

The much-discussed urgency of addressing the obesity epidemic does not obviate the need for well-reasoned actions based on the best available evidence. To the contrary, as underscored by the Institute of Medicine (IOM) report on *Accelerating Progress in Obesity Prevention* — *Solving the Weight of the Nation* (1), the urgency of addressing the epidemic compels actions, often policy-related and for both the short- and long-term, that are feasible, work well, and work together, and that do not waste scarce resources or have unintended adverse consequences. This essay highlights findings and implications of a prior IOM report, *Bridging the Evidence Gap in Obesity Prevention — A Framework to Inform Decision Making* (2), in the view of 2 of the IOM study committee members (Appendix) and a colleague who is involved in evaluation of Kaiser Permanente’s Community Health Initiatives. Below we describe the evidence framework that resulted from the study committee’s consensus process and provide some examples of how it can be applied to evaluate existing evidence and inform the generation of new evidence. 

Decisions about obesity prevention are being made daily in communities, states, and countries worldwide. The *Bridging the Evidence Gap* report explains that timely and credible evidence is needed to help decision makers decide what to do and understand how to do it, distinguish actions that are likely to be effective from those that are not, justify particular actions in high-risk populations, quantify likely effects, estimate costs and cost-effectiveness, set priorities regarding specific outcomes, determine who benefits, and anticipate potential problems. The report sponsors — Kaiser Permanente, The Robert Wood Johnson Foundation, and the Centers for Disease Control and Prevention — were motivated by their perception that effective approaches to obesity prevention were proving difficult to identify, creating a risk that ongoing efforts to address the problem would be ill-conceived or haphazard.

Below we describe the evidence framework that resulted from the study committee’s consensus process and provide some examples of how it can be applied to evaluate existing evidence and inform the generation of new evidence. The full *Bridging the Evidence Gap* report and related summaries, as well as the presentations from 2 workshops convened by the committee, are available from IOM at www.iom.edu/Reports/2010/Bridging-the-Evidence-Gap-in-Obesity-Prevention-A-Framework-to-Inform-Decision-Making.aspx.

## Using Tools From Evidence-Based Public Health

Early in its deliberations, the study committee decided that it would be essential to understand how various forms of evidence are generated and used in obesity prevention efforts and are related to core concepts in the broader sphere of evidence-based public health (EBPH) (2,3). This conclusion was based on a review of the available evidence base for obesity prevention and the judgment that the research approaches being applied, both for specific studies and for evidence synthesis, were framed too narrowly, were inconsistent with respect to how obesity prevention was being conceptualized, and were not focused on the types of intervention or policy questions relevant to obesity prevention in a public health context.

Described by Kohatsu et al, EBPH is a process of integrating science-based interventions with community preferences to improve the health of populations (4). As in evidence-based medicine (EBM), the basic principles of scientific validity apply in EBPH. However, in EBPH, approaches for achieving scientific validity and rigor are broadened to allow for a more balanced consideration of both internal and external validity to assess effectiveness (ie, are results shown as a result of implementing the program) and relevance (ie, can findings be generalized to new settings and populations) in public health contexts, which can be very different from the therapeutic settings addressed in EBM. The potential distinctions can be highlighted by reference to common challenges in evidence-based practice in public health, social work, medicine, nursing, and psychology, as identified by Satterfield et al, related to 1) how evidence should be defined, 2) how and when population-level contextual factors should enter the decision-making process; 3) the definition and role of the experts or key stakeholders, and 4) what variables should be considered when selecting an evidence-based practice (eg, age, social class) (5). The IOM study committee considered each of the challenges within the specific context of obesity prevention, and the L.E.A.D. framework provides specific guidance about how to address them.

To align with the core concepts of EBPH, approaches and tools should be geared to the types of research and practice issues that arise in public health (6,7). For example, in obesity prevention, much of the relevant evidence relates to environmental circumstances and policies that influence the likelihood that people will achieve and maintain food intake and physical activity patterns that prevent or limit excess weight gain. Preventive strategies involving environmental and policy changes are designed to provide opportunities, support, and cues to help people develop healthier behaviors and to make it easier to practice these behaviors. Environmental and policy changes may complement individual-level programs and can benefit all people exposed to the environment rather than focusing on changing the behavior of one person at a time. Alterations in the policy environment may affect behaviors directly (eg, raising the price of sugar-sweetened beverages may decrease consumption) or by altering social norms (eg, worksite policies that promote physical activity may increase physical activity by providing social support) (8).

## The L.E.A.D. Framework Elements: Locate Evidence, Evaluate It, Assemble It, and Inform Decisions

**Figure 1:**
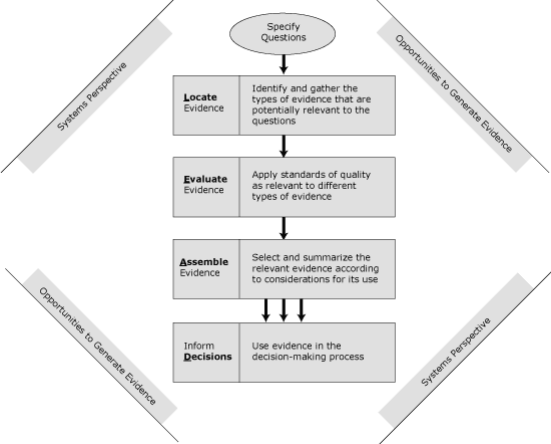
The IOM L.E.A.D. Framework to Inform Decision Making About Obesity Prevention. Adapted from the Institute of Medicine (2).

Although to date few approaches to EBPH have been systems-based, evidence gathering and use in the L.E.A.D. framework has a systems perspective (Figure 1). Precisely because systems approaches may be daunting to researchers and practitioners who have been acculturated to value simplicity and to focus on and isolate specific issues, chapter 4 of the report is devoted to explaining the concepts of systems thinking and how it has been and can be used to inform decisions about obesity prevention. Key messages in that chapter emphasize the importance of addressing the multilevel and dynamic complexity of real-world contexts, attempting to consider the whole picture even when focusing on one aspect, and considering interactions among types and levels of interventions. This perspective helps to anticipate a broader set of outcomes — both positive and adverse — that may be associated with a policy or program and also to see how a particular policy or program might be enhanced or inhibited by others or by situational factors. This, in turn, helps to define what type of information is relevant, whether the information gathered is sufficiently comprehensive, and what its implications are. Also, the use of systems thinking or perspectives in the L.E.A.D. framework does not require the use of mathematical modeling approaches, simulations, or causal mapping techniques used in formal systems science; however, the potential value of such approaches is recognized and encouraged when appropriate, complementary to (not substitutes for) other types of evidence, and some examples are provided.

### Specifying Questions

**Figure 2:**
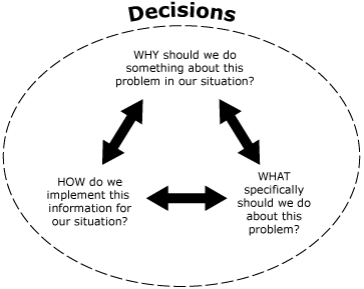
Questions that guide the gathering of evidence. Adapted from the Institute of Medicine (2).

The L.E.A.D. framework adapts an EBPH typology recommended by the International Obesity Task Force (3,9) for specifying questions (Figure 2). The “why” questions help decision makers frame reasons for considering or taking an action based on issues in their specific locality, region, or situation, which may include posing questions to assess baseline status or resources of the relevant population or setting. The “what” questions focus on selection of specific programmatic or policy initiatives and may include assessments of the potential effectiveness or value of approaches designed for specific settings (eg, schools, worksites, faith organizations) or subpopulations (eg, children of different ages, ethnic minority populations, low-income populations). “How” questions prompt for information about implementation issues, including resources required, how effects can be sustained over time, and factors that determine the generalizability or transferability of an approach tested in one setting to another setting.

### Locating and Evaluating Evidence

The L.E.A.D. framework identifies several different types of evidence and study designs that can be useful for informing decisions about obesity prevention and provides resources and explanations in the report narrative and appendices. L.E.A.D. does not imply lowering standards for the quality of evidence used in public health compared with medicine. Rather, it introduces the concept that broader and different standards are needed to account for the complexity and practicalities associated with issues that arise in obesity prevention and other public health problems (eg, tobacco use, environmental health issues). Evidence sources may be quantitative or qualitative or a combination of both and may come from academic research, program evaluations, surveys, polls, reports, or policy documents related to obesity or to other public health issues from which parallels can be drawn. Specific study designs and methods identified in the report include experiments and quasi-experiments, qualitative research, mixed methods, evidence synthesis methods, parallel evidence, and expert knowledge. The point is to be systematic but much more inclusive when determining what constitutes useful evidence related to a particular question. Evidence quality is then to be evaluated by standards appropriate to that type of evidence rather than by a single standard. Examples of existing criteria for assessing quality of evidence from these different methods are provided.

One way that EBPH differs from EBM is that it relies less on randomized controlled trials and more on approaches that assess external validity. Study design cannot be the sole criterion for whether evidence is useful. A randomized controlled trial is the most rigorous design for hypothesis testing (10) but is not feasible for many examples of obesity interventions because the evaluator cannot randomly assign exposure (eg, a policy). Randomized designs also may provide incomplete information if the experiments evaluate artificial scenarios that have limited or only partial relevance to what happens in reality. Partial relevance might occur if the trial manipulates only 1 or 2 of several variables that interact in a dynamic manner to affect an outcome. In general, studies of obesity prevention have tended to overemphasize internal validity (eg, well-controlled efficacy trials) while giving sparse attention to external validity (eg, the translation of science to the various circumstances of practice) (11,12). The work of Klesges et al shows that some contextual variables (eg, cost, program sustainability) are missing entirely in the peer-reviewed literature on obesity prevention (13). Conversely, rigorous evaluations of nonrandomized, “natural experiments” can be informative for many obesity prevention questions. Natural experiments refer to naturally occurring circumstances in which different populations are exposed or not exposed to a potentially causal factor (eg, a stringent new school food policy) such that the situation resembles a true experiment in which study participants are assigned to exposed and unexposed groups (14). These types of studies often involve “messier” study designs (ie, complex, multilevel, multisector interventions) and suggest the need to take a broader perspective in identifying evidence and pay greater attention to external validity and situational or population-specific variables. The real world is actually “messy” or, more formally, complex. In a natural experiment, the strongest design possible (internal validity) is essential, and elements of external validity must be addressed. The L.E.A.D. framework guidance is designed to help incorporate this complexity into evidence rather than controlling for it, which detracts from and may completely remove contextual relevance.

### Assembling Evidence and Informing Decisions

The ultimate goal of the L.E.A.D. framework process is to assemble evidence in a way that is useful to decision makers. The framework recommends a standard template that can be used to report results to decision makers, which prompts for 1) a statement of the question, 2) a transparent description of the strategy used to locate the evidence, 3) a table reporting the evidence, and 4) a summary of the evidence organized as answers to the EBPH-derived questions (Figure 2). Because policy questions often focus on selecting the most feasible intervention, especially detailed guidance is provided about how to interpret and assemble evidence related to “what” questions. This guidance includes a discussion of how one might apply theory or program logic and a systems lens in interpreting evidence, considerations for weighting different types of evidence, and potential ways to blend information from disparate sources and to evaluate effects. Potentially useful tools and frameworks for grading and assembling different types of evidence are identified and include tools used in EBM: meta-analytic approaches to determine intervention effect size and the GRADE (Grading of Recommendations Assessment, Development, and Evaluation) system for evaluating factors affecting the strength of recommendations. Other tools identified of particular relevance to public health applications include realist reviews that use mixed methods to assess intervention effectiveness, the systematic review approach of the *Guide to Community Preventive Services* framework for translating evidence into recommendations, the RE-AIM framework (Reach, Effectiveness, Adoption, Implementation, and Maintenance) for translating research into practice, the Health Canada risk assessment and management framework, the International Obesity Task Force obesity prevention portfolio approach for selecting a set of interventions, and the Green and Kreuter framework for identifying program components and interventions (“matching, mapping, pooling, and patching”) (9,15-19).

In addition to the systems perspective, recognizing opportunities to generate new evidence is also recommended as a theme of the L.E.A.D. framework. Such opportunities might arise at any stage of the process. Generation of new evidence is critical not only because of the dearth of suitable evidence about obesity-relevant environmental and policy changes but also to keep the evidence base current with the dynamics of the obesity problem. The “why,” “what,” and “how” questions in Figure 2 should guide the type of evidence generated. EBPH approaches to filling evidence gaps include program evaluations and natural experiments. This is termed “practice-based evidence” and may also include pre-evaluations or “evaluability assessments” of promising programs and continuous quality improvement. The case study (Box) of data collection in support of school-based physical activity interventions by evaluating programs in northern California communities illustrates the L.E.A.D. concept of using practice settings to increase the evidence base.

**Box. L.E.A.D. Framework Case Study**

**Kaiser Permanente Healthy Eating Active Living Community Health Initiative (HEAL-CHI)**

**Background**

Obesity is a major health problem among both adults and children in the United States (20,21). In California, more than 60% of adults are obese or overweight (22). Being either obese or overweight increases the risk for many chronic diseases (eg, heart disease, type 2 diabetes, certain cancers, stroke) (23). The prevalence of obesity is higher in ethnic minority populations compared with non-Hispanic whites. Moreover, progress being made in curbing the epidemic may not reach all groups equally. The prevalence of high body mass index in California children is declining in some groups but remains high and is not declining among American Indians and African Americans (24).

**Context**

Reversing the obesity epidemic requires a sustained effort at multiple levels, including environmental and policy changes (25). Since children spend a large fraction of their day at school, schools offer a promising environment for intervention. Recommended school strategies include increasing healthier food choices, and increasing the amount of time spent in physical education classes (25,26). Numerous communities in the Kaiser Permanente (KP) Community Health Initiatives (27) have implemented school-based programs targeting either food or physical activity behaviors. This case study focuses specifically on attempts to increase physical activity through in-school or after-school physical activity programs in the 3 communities in the KP Northern California HEAL-CHI initiative.

**Evidence Base**

School physical education programs are one of the few areas in environmental obesity prevention where the Centers for Disease Control and Prevention (CDC) *Guide to Community Preventive Services* has made a positive recommendation (28), on the basis of many evidence-based programs, including the CATCH program (29) that has been widely disseminated. CDC and the Institute of Medicine have also made recommendations that include extracurricular (eg, after school) physical activity programs but the evidence for the effectiveness of those programs is more limited.

As recommended in the L.E.A.D. framework, an approach to increasing the evidence base is to evaluate community interventions that either attempt to implement existing programs such as CATCH or create new programs developed by schools and communities themselves. Although these evaluations do not use experimental designs, they follow the recommendations in the LE.A.D. report that advocate for taking advantage of all opportunities to increasing the evidence and exploring alternative, nonexperimental research designs.

**Lessons and Future Directions**

The HEAL-CHI evaluation used a logic model approach to assessing intervention impact that combined estimates of the reach and strength of the interventions with population-level measures of physical activity, nutrition, and overweight (eg, surveys of youth and adults). In particular, we assessed whether there were significant positive population-level changes where “high-dose” (ie, high reach and strength) interventions were implemented. The information about reach and strength came from independent assessments of the number of people exposed and the intensity of the interventions. For example, one high-dose intervention was an after school physical activity program that one-quarter of all children participated in that added 20 minutes per day of moderate to vigorous physical activity. Results indicated that in almost half of the cases (4 of 9) where high-dose interventions were implemented, significant positive changes favored the intervention. For example, in the community implementing the after-school physical activity program, the percentage of seventh graders doing vigorous physical activity at least 20 minutes per day increased from 61% to 67%, while the percentage in comparison communities declined from 56% to 51%.

**Implications**

The HEAL-CHI initiative used L.E.A.D. thinking at several points — both in applying criteria for interventions and in evaluating the results. Our experience suggests that L.E.A.D. may be a useful approach for incorporating evidence into community-based obesity prevention initiatives. The L.E.A.D. framework encourages both taking a broader view of the existing evidence and using an array of designs in doing evaluations that add to the evidence base. If the L.E.A.D. framework is widely adopted, publishing results such as those we found in the HEAL-CHI initiative in peer-reviewed publications will be easier.

### Implications and Future Directions

The *Bridging the Evidence Gap* report directs recommendations to decision makers in the policy and programmatic arenas as well as to those who fund, generate, and publish evidence about obesity prevention and other complex public health challenges. Central themes are to apply the L.E.A.D. framework as a guide in the use and generation of evidence and to incorporate systems-thinking into research activities. The report also recommends the development of resources to support evidence-based public policy decision making and research, including researcher training, compendiums of knowledge, registries of implementation experience, and guidance on standards for evidence evaluation where they are lacking. The need for a public-private consortium to take up dissemination, support for, and further development of the L.E.A.D. framework is emphasized.

In the approximately 2 years since the *Bridging the Evidence Gap* report was released, it has gained visibility among potential users. The report page on the IOM website has generated more than 17,000 page views. The L.E.A.D. framework has been presented at national and international meetings and cited in journal articles and policy documents. Scanning approximately 40 identified citations indicates that most have involved referencing the report in support of the importance of developing comprehensive multistakeholder and multisectoral strategies, taking a systems perspective, or using expanded approaches to evidence-gathering or choice of study designs. Applications of the framework reflected in published documents include a CDC Division of Nutrition, Physical Activity, and Obesity fact sheet that explains how the framework relates to potential uses of their research and practice-based initiatives, evidence sources, and guidance documents (30); adaptation of L.E.A.D. concepts to describe implications of a systems approach for policy and actions to address the global obesity epidemic (31); use of L.E.A.D. concepts to justify and propose a design for a large-scale demonstration and evaluation of a comprehensive community-based obesity prevention strategy beginning in early life (32); use of L.E.A.D. elements as the primary method for a review of progress made by the food industry, governments, and schools in implementing recommendations of 2 earlier IOM reports on childhood obesity (33,34); and extensive use of L.E.A.D. framework perspectives and evidence review guidance in the IOM report that recommends a set of systems-oriented and interrelated strategies to accelerate progress in obesity prevention (1).

What does this mean for obesity prevention and for advancing appreciation for the science and practice of EBPH? The answer depends on further use of frameworks such as L.E.A.D. In an ideal sense, L.E.A.D. could become a transformative and integrative EBPH paradigm and tool, as intended by the IOM study committee that developed it. The transformational aspect is the positioning of evidence needs in a public health context and demonstrating that rigor and relevance can be achieved using EBPH concepts and tools. Advances in obesity prevention will depend in part on articulating the value of multiple and varied types of information for answering policy and practice questions. If widely adopted and used, L.E.A.D. could become a critical component of identifying and using evidence-informed strategies for achieving national health objectives (35). Such use is likely to better link the practice of public health with the science of public health.

## Committee Members

Shiriki K. Kumanyika (Chair) Professor of Epidemiology and Associate Dean for Health Promotion and Disease Prevention, School of Medicine, University of Pennsylvania Perelman School of Medicine, Philadelphia, PA

David B. Abrams, Director, Schroeder Institute for Tobacco Research and Policy Studies, American Legacy Foundation, Washington, DC; Professor, Department of Health, Behavior and Society, The Johns Hopkins Bloomberg School of Public Health, Baltimore, MD

Ross C. Brownson, Professor of Epidemiology, School of Medicine (Siteman Cancer Center) and George Warren Brown School of Social Work, Washington University in Saint Louis, MO

Frank Chaloupka, Professor of Economics, Director, UIC Health Policy Center, University of Illinois at Chicago

Madhabi Chatterji, Associate Professor of Measurement-Evaluation and Education, Teachers College, Columbia University, New York

Barbara A. Dennison, Director of Policy and Research Translation, Division of Chronic Disease and Injury Prevention, New York State Department of Health, Albany, NY

Christina Economos, New Balance Chair, Childhood Nutrition, Friedman School of Nutrition Science and Policy, Tufts University, Boston, MA

Steven Gortmaker, Professor of Practice of Health Sociology, Department of Society, Human Development, and Health, School of Public Health, Harvard University, Boston, MA

Lawrence W. Green, Professor, Department of Epidemiology and Biostatistics, Helen Diller Family Comprehensive Cancer Center, University of California – San Francisco

Robert A. Hiatt, Professor and Co-Chair of Epidemiology and Biostatistics, Deputy Director, Helen Diller Family Comprehensive Cancer Center, University of California – San Francisco

William Purcell, III Director, Institute of Politics, John F. Kennedy School of Government, Harvard University, Cambridge, MA

Robert Sege, Professor of Pediatrics, Boston University School of Medicine, Boston, MA

Harold Sox, Editor Emeritus, Annals of Internal Medicine, American College of Physicians of Internal Medicine, Philadelphia, PA

Adolfo M. Valadez, Assistant Commissioner, Division of Prevention and Preparedness Services, Texas Department of State Health Services, Austin, TX

Leticia Van De Putte, Senator, Texas State Senate, San Antonio, TX

Stephen G. West, Professor of Quantitative and Social Psychology, Department of Psychology, Arizona State University, Tempe, AZ

Committee Staff

Lynn Parker, Scholar

Leslie J. Sim, Program Officer

Emily Ann Miller, Research Associate

Matthew Spear, Senior Program Assistant

Linda D. Meyers, Director, Food and Nutrition Board
