# Erratum, Vol. 9, July 12 Release

**DOI:** 10.5888/pcd9.12_0157e

**Published:** 2012-07-19

Because of an editing error, we inadvertently omitted text from the article "The L.E.A.D. Framework: Using Tools From Evidence-Based Public Health to Address Evidence Needs for Obesity Prevention." The last sentence of the first paragraph should have read, "This essay highlights the findings and implications of a prior IOM report, *Bridging the Evidence Gap in Obesity Prevention — A Framework to Inform Decision Making* (2), in the view of 2 of the IOM study committee members (Appendix) and a colleague who is involved in evaluation of Kaiser Permanente’s Community Health Initiatives." In addition, the Acknowledgment was omitted. It should have appeared as follows: 

## Acknowledgment

Kaiser Permanente, the Robert Wood Johnson Foundation, and the US Centers for Disease Control and Prevention were the sponsors of the report. Drs Kumanyika and Brownson were members of the IOM study committee. Dr Cheadle is involved in the evaluation of Kaiser Permanente’s Community Health Initiatives. Views expressed in this commentary that are not directly identified as content in the report should be attributed to the authors and may not represent the views of the Institute of Medicine. The authors have no conflicts of interest to declare and did not receive financial support for preparing this essay. This correction was made to our website on July 16, 2012, and appears online at http://www.cdc.gov/pcd/issues/2012/12_0157.htm. We regret any confusion or inconvenience this error may have caused. 

